# Physiological assessment of orthostatic intolerance in chronic fatigue syndrome

**DOI:** 10.1186/s12967-022-03289-8

**Published:** 2022-02-16

**Authors:** Benjamin H. Natelson, Jin-Mann S. Lin, Michelle Blate, Sarah Khan, Yang Chen, Elizabeth R. Unger

**Affiliations:** 1 Department of Neurology, Icahn School of Medicine at Mount Sinai, 1468 Madison Avenue, New York, NY 10029 USA; 2 Division of High-Consequence Pathogens and Pathology, Centers for Disease Control and Prevention, National Center for Emerging and Zoonotic Infectious Diseases, 1600 Clifton Road, Atlanta, GA 30333 USA

## Abstract

**Background:**

Orthostatic intolerance-OI is common in Myalgic Encephalomyelitis/Chronic Fatigue Syndrome-ME/CFS. We used a 10-min passive vertical lean test as orthostatic challenge-OC and measured changes in vitals and end tidal CO_2_ (eTCO2). An abnormal physiologic response to OC was identified in 60% of the 63 patients evaluated from one to three times over several years. Hypocapnia, either resting or induced by OC, was the most frequent abnormality, followed by postural orthostatic tachycardia.

**Objective:**

Evaluate the physiologic response of patients with ME/CFS to a standardized OC.

**Design:**

Respiratory and heart rate, blood pressure and eTCO_2_ were recorded twice at the end of 10-min supine rest and then every minute during the 10-min lean. Hypocapnia was eTCO_2_ ≤ 32 mmHg. Orthostatic tachycardia was heart rate increase ≥ 30 beats per minute compared with resting or ≥ 120 BPM. Orthostatic hypotension was decreased systolic pressure ≥ 20 mmHg from baseline. Tachypnea was respiratory rate of  ≥ 20 breaths per minute—either supine or leaning. Questionnaire data on symptom severity, quality of life and mood were collected at visit #2.

**Patients:**

63 consecutive patients fulfilling the 1994 case definition for CFS underwent lean testing at first visit and then annually at visit 2 (n = 48) and 3 (n = 29).

**Measures:**

Supine hypocapnia; orthostatic tachycardia, hypocapnia or hypotension.

**Results:**

The majority of ME/CFS patients (60.3%, 38/63) had an abnormality detected during a lean test at any visit (51%, 50% and 45% at visits 1, 2 and 3, respectively). Hypocapnia at rest or induced by OC was more common and more likely to persist than postural orthostatic tachycardia. Anxiety scores did not differ between those with and without hypocapnia.

**Conclusions:**

The 10-min lean test is useful in evaluation of OI in patients with ME/CFS. The most frequent abnormality, hypocapnia, would be missed without capnography.

## Background

Myalgic encephalomyelitis/chronic fatigue syndrome [ME/CFS] is a medically unexplained illness characterized by fatigue severe enough to produce a substantial decrease in activity. In addition, in its 2015 review of the known science regarding this disorder, the Institute of Medicine recommended the clinical case definition include unrefreshing sleep and post-exertional malaise (minimal exertion producing subsequent, often delayed syndromic worsening) along with either cognitive problems or orthostatic intolerance [1]. The latter symptom is described by the patient as a worsening of symptoms or dizziness while standing or sitting upright.

Orthostatic intolerance [OI] often reflects altered autonomic activity and may be evaluated by measuring heart rate and blood pressure during a 10-min period of passive vertical leaning (the “lean test”). Two commonly reported abnormalities are orthostatic hypotension and postural orthostatic tachycardia syndrome (POTS). However, adding capnography to the measures in the lean test will allow detection of the postural orthostatic syndrome of hypocapnia [POSH], another important cause of orthostatic intolerance. Both POTS and POSH have been recognized for some time [2, 3], and we have found POSH to occur more frequently than any of the other abnormalities [4]. Even in the absence of an orthostatic challenge (i.e., while seated or supine), persons with ME/CFS have been reported to have significantly lower measures of end tidal CO_2_ than healthy controls [5–7].

It is not clear if seated/supine hyperventilation affects whether abnormalities will occur following orthostatic challenge. In addition, there is limited information about whether abnormalities detected during the lean test will persist over time. The purpose of this report is to provide data on both these questions using a sample of patients who attended up to three annual visits related to their illness. This is the first study to look across time at OI in ME/CFS.

## Methods

The study includes 63 consecutive patients who were evaluated and met criteria for chronic fatigue syndrome at the Pain & Fatigue Study Center, Mount Sinai Beth Israel in New York City between 2012 and 2015. All were evaluated by a skilled medical professional who determined they fulfilled criteria for the diagnosis of CFS using the 1994 case definition [8]. They had blood drawn to rule out common medical causes of fatigue such as hypothyroidism, liver abnormalities, Lyme disease, anemia and inflammatory processes. Thus, they had no untreated medical or psychiatric conditions explaining their symptoms and had a long duration of fatigue resulting in a substantial decrease in activity across a number of life spheres accompanied by the presence of at least four of 8 symptoms [sore throat, tender lymph nodes, headache, myalgia, arthralgia, unrefreshing sleep, post-exertional malaise, cognitive problems with attention or memory]. An additional requirement for study entry was that patients had to endorse at least three of these symptoms as substantial or worse [on a scale of mild, moderate, substantial, severe, very severe] and one as at least moderate. Patients were also evaluated to determine if they fulfilled case definitions for fibromyalgia [FM], irritable bowel syndrome [IBS] and multiple chemical sensitivity [MCS] using accepted criteria as previously described [9].

The lean test was performed using a protocol developed by NASA [10]. Specifically, after at least 10 min lying supine, baseline physiological measures—blood pressure, heart and respiratory rates and end tidal CO_2_ [eTCO_2_] as assessed using an Oridion Microstream Capnograph (Medtronics, Inc., Minneapolis, MN)—were recorded twice, a minute apart. Patients were then asked to stand with legs together approximately 6–8 inches from a wall and then to lean for 10 min while physiological measurements were taken every minute. The lean test was repeated at visits of 8 to15-month intervals for up to three visits. After the initial visit, the Structured Clinical Interview for DSM-IV psychiatric diagnoses was administered over the phone. Patients completed questionnaires concerning overall well-being, physical and mental functioning [Short Form Health Survey (SF-36v2) [11]; Multidimensional Fatigue Inventory [12]; CDC symptom inventory [13]; Generalized Anxiety Disorder 7-item questionnaire (GAD-7 [14])] during the second clinical visit. Dizziness severity was assessed using the DePaul Symptom Questionnaire and was quantified by multiplying frequency times intensity [15].

Hypocapnia was defined as eTCO_2_ ≤ 32 mmHg in at least one measure: during supine rest and persisting through orthostatic challenge (supine hypocapnia) or only during orthostatic challenge (postural orthostatic syndrome of hypocapnia or POSH). Postural orthostatic tachycardia syndrome or POTS was defined as a heart rate increase during orthostatic challenge of ≥ 30 beats per minute (BPM) compared with resting or ≥ 120 BPM. Orthostatic hypotension (OH) was defined as a drop of systolic pressure ≥ 20 mmHg from baseline levels occurring on at least 2 determinations after leaning. Tachypnea was defined as a respiratory rate of 20 or higher breaths per minute—either supine or leaning.

Norm-based T-scores (standardized to the 2009 US general population with a mean of 50 and a standard deviation of 10) were calculated for SF-36 subscales, Physical Component Summary (PCS) and Mental Component Summary (MCS). Higher scores represent better functioning. The GAD-7 summed scores range from 0 to 21, with scores ≥ 10 for moderate-to-severe symptom experiences. Scores on each of these tests were compared to the results of physiological monitoring. A fisher’s exact test set at p < 0.05 was used to determine if differences in GAD-7 existed between those patients manifesting supine hypocapnia and those with no abnormality. Potential confounders such as co-morbidity, were used in the logistic regression to examine the likelihood of having a lean test abnormality.

The study was performed with informed consent for medical record abstraction and performance of the NASA lean test as part of protocols reviewed and approved by Mount Sinai Beth Israel IRB and the Centers for Disease Control and Prevention IRB.

## Results

The mean age of the 63 patients was 48.1 years; most were female (82.5%), white (79.3%) and college graduates (84.1%; Table 1). The patients had been ill an average of 14 years. An abnormality during a lean test at any visit [63 patients came for one visit, 48 for two and 29 for three visits] was detected in in 60.3% (38/63), i.e., supine hypocapnia [Supine ↓CO_2_], orthostatic hypotension [OH], postural orthostatic tachycardia syndrome [POTS], or postural orthostatic syndrome of hypocapnia [POSH]. The only demographic variable that differed significantly between those with and without a lean test abnormality was a higher proportion not currently married in the group without lean test abnormality. Sudden onset of CFS was the only illness characteristic that differed significantly between the two groups, being more common among those with a lean test abnormality (57%) than those without (26%).

**Table 1:** Sample characteristics overall and by detection of any lean test abnormality^a^

	Any abnormality (n = 38)	No abnormality (n = 25)	Total (n = 63)
Age (years) (Mean ± SD)	47.1 (± 11.3)	49.7 (± 9.1)	48.1 (± 10.5)
Sex (% female)	84%	80%	83%
Race (% White)	84%	72%	79%
College graduate (%)	84%	84%	84%
Currently insured	92%	92%	92%
Not currently married* (%)	55%	80%	65%
Not employed (%)	66%	56%	62%
Current Psych Dx (%)	26.5%	23.8%	25.4%
Onset status*			
Sudden (%)	57%	26%	45%
Gradual (%)	43%	74%	55%
CDC-SI (Mean ± SD)			
Duration of Illness (Fatigue), years	12.8 (9.1)	16.3 (11.4)	14.2 (10.1)
# of CFS Symptoms	6.4 (2.0)	6.3 (1.5)	6.3 (1.8)
CFS Symptom Summary Score	67.4 (24.8)	65.9 (20.1)	66.8 (22.9)
MFI-20 (Mean ± SD)			
General Fatigue	18.6 (2.1)	17.9 (2.2)	18.3 (2.2)
Physical Fatigue	18.0 (2.5)	16.6 (3.5)	17.4 (3.0)
Mental Fatigue	15.6 (4.0)	14.6 (3.6)	15.2 (3.9)
Reduced Activity	17.0 (3.3)	14.9 (4.3)	16.2 (3.8)
Reduced Motivation	13.5 (4.4)	11.6 (4.1)	12.8 (4.4)
SF-36 T-Scores (Mean ± SD)			
Physical Functioning	31.4 (9.1)	34.7 (8.7)	32.7 (9.0)
Role Physical	23.1 (7.9)	26.1 (10.5)	24.3 (9.0)
Bodily Pain	35.5 (8.9)	34.2 (9.00)	35.0 (8.9)
General Health	29.0 (8.9)	33.1 (11.4)	30.6 (10.0)
Vitality	28.8 (7.7)	30.5 (6.2)	29.5 (7.1)
Social Functioning	22.7 (9.2)	26.5 (7.6)	24.2 (8.7)
Role Emotional	45.2 (13.6)	46.4 (12.4)	45.7 (13.1)
Mental Health	44.9 (10.3)	46.4 (9.5)	45.5 (10.0)
GAD-7 Score (Mean ± SD)	4.1 (4.4)	5.6 (5.1)	4.7 (4.7)
Co-morbidity			
Irritable Bowel Syndrome (IBS)	71%	72%	71%
Fibromyalgia (FM)	29%	16%	24%
Migraine	66%	68%	67%

*p < 0.05

^a^Any Orthostatic Abnormality was defined as postural orthostatic syndrome of hypocapnia, supine hypocapnia, postural orthostatic tachycardia syndrome or orthostatic hypotension detected in at least one visit

The pattern of lean test abnormalities detected at each of the 68 visits among the 38 patients with an abnormal result is shown in Table 2. Among the 38 with abnormalities, 6 did not return for the second visit and an additional 13 did not return for the third visit. An abnormality was not noted at the first visit for 6 of those eventually detected with an abnormality and for 3 of these patients an abnormality was not detected until the third visit. Percentages of patients with abnormalities were similar across the three visits—51%, 50% and 45% respectively. Of the 25 without any abnormality across visits, 9 had only one visit, 6 had two and 10 had 3 visits.

**Table 2:** Abnormalities identified across visits (n = 38 patients with abnormality at any time)

First visit	Second visit	Third visit
Normal	Normal	POSH
Normal	Normal	OH
Normal	Normal	Supine ↓CO_2_
Normal	POSH	
Normal	POSH	
Normal	POTS	Normal
POSH	Normal	Normal
POSH	Normal	Normal
POSH	Normal	
POSH	Normal	Normal
POSH	POSH	Normal
POSH	POSH	
POSH	POSH	Supine ↓CO_2_
POSH	OH	Normal
POSH	POTS/POSH/OH	POTS/POSH
POSH	Supine ↓CO_2_	
POSH	Supine ↓CO_2_/OH	
POSH		
POSH		
POSH		
POSH		
POSH		
POTS	Normal	
POTS	POSH	POSH
POTS	OH	Normal
POTS		
POTS/POSH	POSH	POSH
POTS/POSH	POSH	
POTS/POSH	POTS	POSH
POTS/POSH	Supine ↓CO_2_ /POTS	POTS/POSH
Supine ↓CO_2_	POSH	
Supine ↓CO_2_	POSH	
Supine ↓CO_2_	POSH	
Supine ↓CO_2_	Supine ↓CO_2_	
OH	POSH/OH	
POTS/POSH/OH	Supine ↓CO_2_	Supine ↓CO_2_/OH
Supine ↓CO_2_/OH	POSH	POTS/POSH
Supine ↓CO_2_/OH/POTS	POSH	POSH

POSH, postural orthostatic syndrome of hypocapnia; Supine ↓CO_2_, supine hypocapnia; POTS, Postural orthostatic tachycardia syndrome; OH, Orthostatic hypotension

More than one abnormality was detected in nearly a fourth of the abnormal tests (22.1%, 15 of 68; Table 2). The overlap in abnormalities makes description of the pattern complex. Strikingly, over 80% of the abnormal tests detected hypocapnia either after orthostatic challenge (44 instances) or also while supine (additional 14 instances; total 58 of 68 abnormal tests—85.2%). By contrast, POTS was identified in only 17 abnormal tests (17/68, 25%) and in 11 of these instances was associated with either postural or Supine ↓CO_2_ (including 5 instances with orthostatic hypotension). Only 11 instances of orthostatic hypotension were identified (11/68, 16.1% of abnormal tests) and 8 of these were found in combination with other abnormalities. Among the 26 patients with two visits and an abnormality detected at visit #1, 21 had a persisting abnormality, but the specific diagnosis was maintained for only 4 of those. However, as all instances of Supine ↓CO_2_ persisted during the leaning phase of the test (the orthostatic challenge), if Supine ↓CO_2_ is grouped with POSH as hypocapnia, 16 patients had persistent hypocapnia.

### Relation of supine hypocapnia to supine tachypnea

Based on data from all three visits, of the 11 patients (14 instances) with at least one instance of Supine ↓CO_2_, only one was also tachypneic; of the 31 patients who showed POSH on at least one occasion, three had supine tachypnea; five of these patients had normal breathing while supine but became tachypneic during the lean. Six other patients also developed tachypnea during the lean but did not show POSH.

### Relation to psychiatric diagnosis or GAD-7 scores

Percentages of patients with Supine ↓CO_2_, POSH and/or POTS did not differ among those classified as having a current psychiatric diagnosis or never having had such a diagnosis (Table 1). Also, there was no difference in GAD-7 anxiety scores between those with an abnormality in a lean test and those without; moreover, there was no difference in these scores between those with and without Supine ↓CO_2_ and between those with Supine ↓CO_2_ and those with no orthostatic abnormality.

### Relation to other variables

There was no consistent relation between the number of comorbid, other medically unexplained illnesses (see our earlier report on frequency of these occurring [9]) and response to the lean test. No significant difference was found in the presence of co-morbidity such as irritable bowel syndrome (IBS) (71% vs. 72%), fibromyalgia (FM) (29% vs. 16%), and migraine (66% vs. 68%) between patients with a lean test abnormality and those with no lean test abnormality. Patients with a lean test abnormality had marginally significant lower values than those with no lean test abnormality in SF-36 –Social Functioning (23.0 vs. 27.5, p = 0.082). None of the MFI-20 subscale scores differed. While none of the CDC symptom inventory item scores differed between patients with and without a lean test abnormality, the DePaul dizziness score was higher among those with an abnormality (3.3 vs. 1.5, p = 0.016).

When further adjusting co-morbidities (IBS, FM, and migraine) for the likelihood of having a lean test abnormality, no significant association was found (IBS: adjusted odds ratio (AOR) = 0.84 (0.264 2.646); FM: AOR = 2.21 (0.600 8.152); migraine: AOR = 0.96 (0.322 2.876)).

## Discussion

This report serves as a replication and extension of earlier important work in which abnormalities of breathing have been reported to be common in ME/CFS [16]. In this study, we used repeated assessments of physiologic responses to a 10 min lean test—an orthostatic challenge—in patients with CFS and found that most (60.3%, 38/63) had an abnormality on at least one such test. This series of evaluations revealed several striking results. First, the postural orthostatic syndrome of hypocapnia or hyperventilation [POSH] is much more prevalent than the postural orthostatic tachycardia syndrome [POTS] or orthostatic hypotension—an outcome which we [4] and Naschitz [16] have previously noted. This result should encourage practitioners who care for ME/CFS patients to purchase the required capnograph to allow assessment of breathing rates and eTCO_2_. Our definition of hypocapnia, one value at or less than 32 mmHg, is conservative given that 45 healthy controls had a mean eTC0_2_ of 40.8 mmHg ± 0.3 mmHg SD [16].

Tachypnea has been reported to be common in ME/CFS [17], but we did not find this to be the case among the 32 patients with physiological evidence of orthostatic intolerance on Visit #1. Importantly, capnography can confirm that the reductions in exhaled CO_2_ are not usually a function of tachypnea but instead occur due to hyperpnea inferred from the normal respiratory rates. Having this information should allow health care providers to work with respiratory therapists to try to reduce depth of breathing while standing using biofeedback techniques or by increasing respiratory effort as can occur while wearing an N-95 mask or some other device that makes breathing harder. Since orthostatic intolerance often follows hypovolemia [18], increasing blood volume by using oral rehydrating salts [19] and compression hose [20] can also help.

The second finding of interest had to do with the tendency for hypocapnia to persist either as Supine ↓CO_2_ or to change to POSH. Five of six patients with supine hypocapnia at Visit #1 had POSH at visit #2, and one continued to show Supine ↓CO_2_. Five patients with POSH or POSH + POTS at Visit #1 had Supine ↓CO_2_ at Visit #2; and, a fifth who had POSH in Visits 1 and 2, displayed Supine ↓CO_2_ on Visit #3. While a standard teaching in medical texts is that hypocapnia in an otherwise normal person indicates underlying anxiety, we found no difference in the Generalized Anxiety Disorder 7-item questionnaire between patients with and without hypocapnia.

Reasons for hypocapnia in patients with ME/CFS are not known, but we suggest several possibilities. Hypocapnia could be a direct physiological consequence of hypovolemia that is known to occur in ME/CFS [21]. In addition, substantial hypovolemia could alter ventilation:perfusion in parts of the lungs rendering eTCO2 assessment inaccurate. Supine ↓CO_2_ could be a physiological manifestation of gravitational stress or could reflect anxiety that was not captured in the methods used. Further research will be in order to determine the role of each of these possibilities. Interestingly, sudden onset of illness, suggested to be due to presumed infectious trigger, was more common among the patients with an abnormality detected in a lean test. It is worth exploring whether orthostatic abnormalities could be used to identify subgroups of patients with ME/CFS who may have similar underlying causes. Naschitz et al. have found different responses to orthostatic challenge between ME/CFS and fibromyalgia [22]. Determining if the range of abnormalities noted here occur more frequently in the presence of other comorbid conditions is a question for future research.

This study has several limitations, including number of patients, one recruitment site, restricted demographic representation, long duration of illness, and use of a device measuring eTCO2 rather than PaCO2. Another limitation is that we determined psychiatric status and level of anxiety on only one visit. Nonetheless, these data support earlier work that a subgroup of patients with ME/CFS have physiological abnormalities as assessed by an orthostatic challenge. As expected, these patients report more dizziness than those without these abnormalities. Because nearly half of all patients tested had evidence of orthostatic intolerance, it is important that testing for this problem including the use of capnography be a routine part of the evaluation to identify both supine and orthostatic hypocapnia—the most common manifestations of orthostatic intolerance found. While the majority of patients showing orthostatic intolerance on Visit #1 continued to do so on subsequent visits, there was variability in the type of lean test abnormalities in repeat visits, and some patients with normal lean test results subsequently developed them. Given the low cost and ease of incorporation of the lean test into the clinic routine, it may be helpful to include it at each visit. Further work is needed to determine how well the results of lean testing correlate with symptomatic orthostatic intolerance and whether the presence of such abnormalities could be used to monitor the response to therapy.

## Data Availability

Contact authors.

## References

[1] IOM. Beyond Myalgic Encephalomyelitis/Chronic Fatigue Syndrome: Redefining an Illness. Washington (DC); 2015.

[2] Schondorf R, Low PA (1993). Idiopathic postural orthostatic tachycardia syndrome: an attenuated form of acute pandysautonomia?. Neurology.

[3] Naschitz JE, Rosner I, Rozenbaum M (2000). The capnography head-up tilt test for evaluation of chronic fatigue syndrome. Semin Arthritis Rheum.

[4] Natelson BH, Intriligator R, Cherniack NS (2007). Hypocapnia is a biological marker for orthostatic intolerance in some patients with chronic fatigue syndrome. Dyn Med.

[5] Lavietes MH, Natelson BH, Cordero DL (1996). Does the stressed chronic fatigue syndrome patient hyperventilate?. Int J Behav Med.

[6] Novak P (2018). Hypocapnic cerebral hypoperfusion: A biomarker of orthostatic intolerance. PLoS ONE.

[7] Bazelmans E, Bleijenberg G, Vercoulen JH (1997). The chronic fatigue syndrome and hyperventilation. J Psychosom Res.

[8] Fukuda K, Straus SE, Hickie I (1994). The chronic fatigue syndrome: A comprehensive approach to its definition and study. Ann Intern Med.

[9] Natelson BH, Lin JMS, Lange G (2019). The effect of comorbid medical and psychiatric diagnoses on chronic fatigue syndrome. Ann Med.

[10] Bungo MW, Charles JB, Johnson PC (1985). Cardiovascular deconditioning during space flight and the use of saline as a countermeasure to orthostatic intolerance. Aviat Space Environ Med.

[11] Ware JE, Snow KK, Kosinski M, et al. SF-36 health survey. Manual and interpretation guide. 1 ed. Boston: The Health Institute, New England Medical Center ;1993.

[12] Vercoulen JHMM, Hommes OR, Swanink MA (1996). The measurement of fatigue in patients with multiple sclerosis - A multidimensional comparison with patients with chronic fatigue syndrome and healthy subjects. Arch Neurol.

[13] Wagner D, Nisenbaum R, Heim C (2005). Psychometric properties of the CDC Symptom Inventory for assessment of chronic fatigue syndrome. Popul Health Metrics.

[14] Spitzer RL, Kroenke K, Williams JB (2006). A brief measure for assessing generalized anxiety disorder: the GAD-7. Arch Intern Med.

[15] Jason LA, Evans M, Porter N (2010). The development of a revised canadian myalgic encephalomyelitis chronic fatigue syndrome case definition. Am J Biochem Biotechnol.

[16] Naschitz JE, Mussafia-Priselac R, Kovalev Y (2006). Patterns of hypocapnia on tilt in patients with fibromyalgia, chronic fatigue syndrome, nonspecific dizziness, and neurally mediated syncope. Am J Med Sci.

[17] Cotler J, Katz BZ, Reurts-Post C (2020). A hierarchical logistic regression predicting rapid respiratory rates from post-exertional malaise. Fatigue.

[18] Garland EM, Celedonio JE, Raj SR (2015). Postural tachycardia syndrome: beyond orthostatic intolerance. Curr Neurol Neurosci Rep.

[19] Medow MS, Guber K, Chokshi S (2019). The benefits of oral rehydration on orthostatic intolerance in children with postural tachycardia syndrome. J Pediatr.

[20] Schondorf R, Benoit J, Stein R (2005). Cerebral autoregulation is preserved in postural tachycardia syndrome. J Appl Physiol.

[21] Hurwitz BE, Coryell VT, Parker M (2009). Chronic fatigue syndrome: illness severity, sedentary lifestyle, blood volume and evidence of diminished cardiac function. Clin Sci.

[22] Naschitz JE, Rozenbaum M, Rosner I (2001). Cardiovascular response to upright tilt in fibromyalgia differs from that in chronic fatigue syndrome. J Rheumatol.

